# Time Spent on Medical Round Activities, Distance Walked, and Time-Motion in the General Medicine Department at Hamad General Hospital in Qatar

**DOI:** 10.7759/cureus.37935

**Published:** 2023-04-21

**Authors:** Anas Al Halabi, Elmukhtar Habas, Khalifa L Farfar, Hafedh Ghazouani, Gamal Alfitori, Moza A Abdulla, Abdelsalam M Borham, Fahmi Y Khan

**Affiliations:** 1 Quality and Patient Safety, Hamad Medical Corporation, Doha, QAT; 2 Internal Medicine, Hamad General Hospital, Doha, QAT; 3 Internal Medicine, Al Wakra Hospital, Al Wakra, QAT

**Keywords:** round time, qatar, gim, medical round, hgh, walking distance

## Abstract

Background

The daily morning round is a routine activity performed by medical teams. During the morning round, updates on the patient’s clinical condition, new laboratory results, and other test results are reviewed and discussed between team members, the patient, and at times the family. Completing these tasks takes time. The design of the patient location differs between hospitals, and significant distance between patients can considerably affect round times. This study assesses physicians’ time spent on clinical activities, the distance traveled, and the time they spend walking between patients during daily morning rounds to identify better reorganization methods to reduce wasted time.

Methodology

The survey was self-administered and had no intervention needing ethical approval. The research team’s leader engaged two observers (a general practitioner from another department and a general internal medicine department case manager) to collect the data. The general practitioner was a medical graduate doctor, while the bed manager was not a medical college graduate. They observed 10 rounds over 10 non-consecutive days from July 1 to July 30, 2022. They recorded daily activities during the daily morning round, including time spent with patients, family conversations, bedside education, medication, social issues, and the time and distance required to move from patient to patient and from one location to location. The informal conversations about age, work history, and other small talk were recorded and converted into quantitative data. In each round, records were given to a statistician for rechecking. Subsequently, the records were imported into a Microsoft Excel spreadsheet for further statistical analysis. For continuous variables, the data were summarized as mean, median, and standard deviation. For categorical variables, the data were summarized as counts or proportions.

Results

On average, the duration of the daily morning round was 161.7 ± 17.3 minutes. The average number of patients seen by the general internal medicine round team was 14. The median patient encounter time per patient was 14 minutes (11-19 minutes), with an average of 12 minutes. An average of 8.6 employees participated in the 10-day rounds. The physician spent 41.2% of the time in direct contact with the patient during the morning round, 11.4% in maintaining electronic medical records, and 18.20% in bedside teaching. Additionally, 7.1% of the round time was spent because of interruptions by clinical and non-clinical staff other than team members or family members who were not in the room. Furthermore, a team member walked an average of 763 ± 54.5 m (667-872 m) per round, costing 35.7 minutes (22.1%) of the total round time.

Conclusions

The daily morning round time was significantly longer compared with the reported round times. Relocating patient beds to a common location reduced the rounding time by 22.30%. Disruption, teaching, and medical instruction must also be considered and shortened to reduce the morning round time.

## Introduction

Qatar’s healthcare sector, like other sectors, foresees a future of escalating limitations. Serious difficulties might challenge the sustainability and quality of services, such as longer duration of daily morning rounds. As reported, the daily morning round time is usually between 105 and 122 minutes [1]. The time wasted in unnecessary activities during the round must be reduced to maintain the quality of care given. Mostly, individuals conceive that time waste is a part of working time, although it is not time used for patient care (service production). Most of the wasted time in patient rounds is rooted in clinical practice and models of care. Low-value services and the unnecessary utilization of clinical resources compromise the sustainability of high-value care and prevent the advancement of new interventions [2].

Daily morning rounds are widely recognized as essential to hospital admission management worldwide. Despite reports that the prevalence of daily ward medical rounds is declining [3], ward rounds are still widely conducted [4]. The team stops at each bedside to talk to the patient and see how they are doing. Diagnosis, prognosis, treatment planning, and social issues are the most frequently discussed topics during ward rounds [5]. Research highlights the importance of effective communication, collaboration, and standardization of processes at departmental levels, involving groups of practitioners to discuss, consider, and decide about their patient care [6]. In addition, it has been shown that there are advantages to spending more time with the patient; therefore, time constraints may be a concern [7]. Non-technical skills of practitioners, such as communication, teamwork, and leadership, are also the subject of research [8]. It is well-documented that daily morning rounds have an important influence on patient safety and play a vital role in inpatient quality management [9]. Furthermore, daily round features are an excellent starting point for planning to improve inpatient care [10].

An operational study was conducted in the Medicine Department at Hamad General Hospital (HGH) for patient experience time in the Emergency Department (ED). A study reported that the waiting time could be decreased to almost 10 hours and even six hours if patient locations were in one or two nearby areas with the implanting of the admission team [11]. Because of a lack of beds in the medical wards, patients are taken to the ED, and once a vacant bed is available in the medical wards, patients are moved to four wards at HGH for further care. Throughout the morning visits, physicians may have to traverse far-reaching distances to reach patients’ locations, necessitating stairs or elevators. As they progress, they might be delayed by other healthcare providers and patients or family members for varying reasons (interruption time), affecting a comprehensive patient assessment, satisfactory evaluation, and care. Considering rounds and other services, it is apparent that team members are overloaded, indicating a need to prioritize the tasks and duties, including rounds, teaching, and clinics. These tasks excessively overload the medical team, negatively impacting patient healthcare quality.

Daily round-time loss recognition and correction can improve team members’ concentration, clinical care, and patient safety quality. Hence, this study aims to determine the duration of physicians’ rounds to complete clinical activities, the distance they walked, and the time they spent during daily morning rounds by medical teams in HGH. Identifying these time losses can reduce round duration, leaving more time for direct patient contact and care. Moreover, this study highlights the importance and benefits of daily rounds, which are often undervalued.

## Materials and methods

Using longitudinal, quantitative, direct observation, we describe the daily morning round in the medical department. In this study, the daily morning rounds of 10 different medical subteams were observed by two observers between July 1 and July 30, 2022. This study was conducted over 10 non-consecutive days in the medical department, where the observer conducted rounds with one of the 10 different teams in four wards at HGH and the ED. The observers were a general practitioner and a bed manager who had previously worked as a nurse. Team leaders and members were informed about the observers and their duties.

A stopwatch (chronometer) for time interval measurement and the move’s application (Proto Geos software Navigates) were used on a phone tablet to track and measure the distance and location of the start and end points. Two observers were assigned to observe the morning rounds to assess their activity sequence, time spent in patient care, time spent in moving from one patient to the next, distance traveled, and time elapsed during travel between patients at the same and various locations. After each daily morning round, observers collected the data and entered it into a Microsoft Excel spreadsheet. The data were rechecked by a statistician.

**Figure 1:**
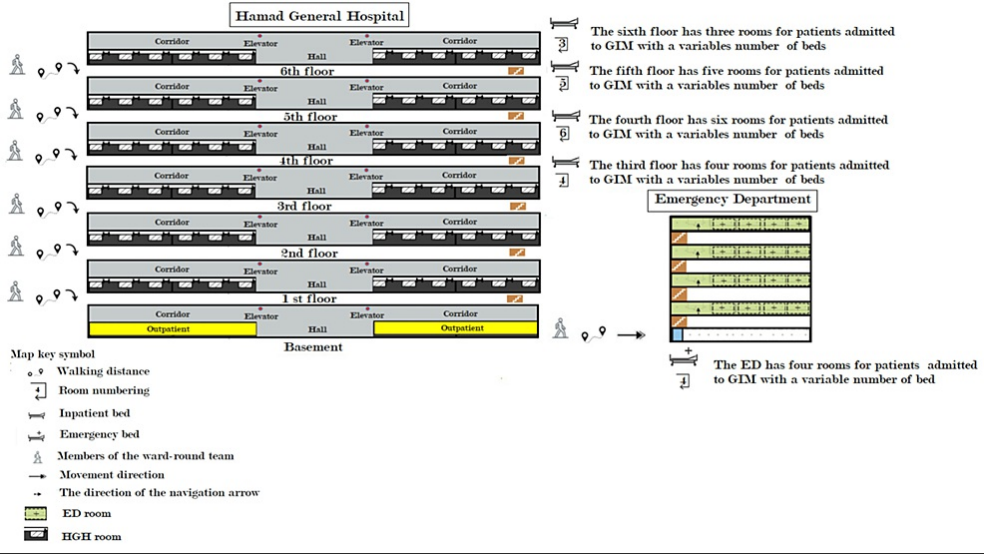
Workplace design. GIM: general internal medicine; ED: emergency department; HGH: Hamad General Hospital

This study focused on general medicine inpatient locations and morning round-time duration at HGH. The general medical department is the admitting department for patients, even from other subspecialties such as nephrology, rheumatology, dermatology, endocrinology, diabetes, and even cardiology patients who present with diseases. We used a chart of workplace design to evaluate the time spent on each activity daily during morning rounds. The study incorporated three distinct architectural units, namely, the *hospital ward*, *ward hallways*, and *hospital passageway*. There were 19 general medicine department inpatient admission locations. To visit these locations, the teams traversed through a track design consisting of six wards (three in HGH and three in the ED), passageways between the HMC of the workplace hospital and ED, and nine ward hallways. Figure 1 illustrates the track design of the workplace.

The workforce of the general medicine department consisted of 35 consultants, nine fellows, and approximately 70 residents. During the study, they were assigned to long-term and general medical ward patient care. The study was conducted in 201 general medicine-assigned beds at the HGH in Qatar. Each subteam consisted of one consultant and sometimes one fellow, four residents, two interns, a clinical pharmacist, a case manager, and one to three students. The subteam covered the care of 15-25 patients (sometimes more) between 7 AM and 3 PM. General internal medicine teams performed ward rounds daily for the whole week, starting at 8 AM to 12 noon or sometimes later, depending on the number and the complexity of patients’ illnesses. The team leader discussed the patient’s care needs with his medical and paramedical staff. The paramedical staff members were physiotherapists and social workers. The team members usually met after the morning report at 8:00 AM after completing the daily morning report and the resident handover for new issues related to the team’s patients by the resident(s) in the evening and night shifts.

Statistical analysis

The observers followed the 10 subteams for a total of 10 visits, i.e., each subteam was observed once to minimize the differences in the care points between the subteams. Leader names, employee names, or their corporation numbers were not collected, reducing the observers’ influence on the team’s activity duration. The observers monitored on-site activity in real-time and recorded the time each team member spent on each activity (e.g., instruction, interviews, bedside tasks, physical examinations, scheduling of goals, organization, planning, and family visits). Each team member also tracked the distance and time walked at each workplace. The observers categorized the behaviors into the following five main aspects: patient care, teaching, electronic health records (EHRs), administration, and social disruptions. To avoid disturbing the team, the observers were silent during the round. Observers left the team and then submitted their data to the statistician, who compiled a report from data collected through a multi-method approach (observation and informal conversation with small talk).

Four elements were involved in this study’s data management and statistical analysis, namely, the parameters, informal group interview, application of the moves, and statistical analysis. First, various measures, such as the distance and duration of the round, were used during the operation design process. Second, our statistician conducted informal interviews and small talk with each team member to determine their age and work experience. The statistician then recorded these conversations and converted them into numbers. Third, we measured the distance between any two points using an online geospatial-driven application called Proto Geos Navigates Software (a geospatial-based online app that provides accurate distances between two points). Then we used a chronometer to measure the time of movement of the place where it started and ended. Finally, the observers entered the data into a Microsoft Excel worksheet. The statistician then checked whether the data met all the requirements. The statistician structured and coded each data point in a Microsoft Excel spreadsheet for further analysis. The data were then analyzed using Minitab Professional Version 13 (Minitab, Inc., State College, PA, USA). For continuous variables, we used measures of central tendency (mean, median, mode) and variability (standard deviation), and for categorical variables, we used frequencies or percentages to measure central tendency.

Ethical approval

Although the study involved humans (doctors, bed managers, and case managers), there were no interventions. Hence, the study did not require ethical approval per Hamad Ethical Committee.

## Results

The number of team members participating in the round and the type of participants differed. In total, the teams consisted of 88 individuals (15 doctors with 10 consultants and five fellows), 16 nurses, 10 case managers, eight social workers, 14 residents, seven different trainee grades (students, graduates), eight interns, 10 clinical pharmacists, with an average of eight years of professional experience and an average age of 36 years. The average number of employees participating in the 10 rounds was 8.6 people/round. The total number of patients examined during the 10 rounds observed (observed over 40.8 hours) was 159. The number of new patients was 64 (40.25%), and the number of follow-up patients was 95 (59.75%), with the median number of patients per round being 14 (12-19 ± 5.38). The average duration of direct patient contact was 12 (range = 8-19, SD = ±7.1) minutes. Table 1 shows the patients and participants observed during the morning rounds.

**Table 1:** Patients and attendees observed during all 10 medical routine morning rounds. ^a^: Data derived from direct observation. ^b^: Data derived from informal interviews with small talk regarding age and work experience.

Staff	Attendees during all 10 medical routine morning rounds
Doctor	15
Nurse	16
Case manager	10
Social worker	8
Resident	14
Intern	8
Stager	7
Pharmacists	10
Total number of attendees	88
Each round’s statistics for attendees^a^	Median = 9
	Minimum = 7
	Maximum = 14
	First quartile = 8
	Third quartile = 10
	Interquartile range = 2
	Standard deviation = 2.87
	Mean = 8.6
Age^a^	Mean = 36 years
Years experience in job position^b^	Mean = 8 years
Patient	Patients viewed during all 10 medical routine morning rounds
Each round’s statistics on patients seen	Median = 14
	Minimum = 11
	Maximum = 19
	First quartile = 12
	Third quartile = 17.25
	Interquartile range = 5.25
	Standard deviation = 5.38
	Mean = 11.75. Mean patient visit duration = 12 minutes (range = 10–15; Z = ±7.1]
	The number of new patients was 64 (40.25%), and the number of follow-up patients was 95 (59.75%)

**Figure 2:**
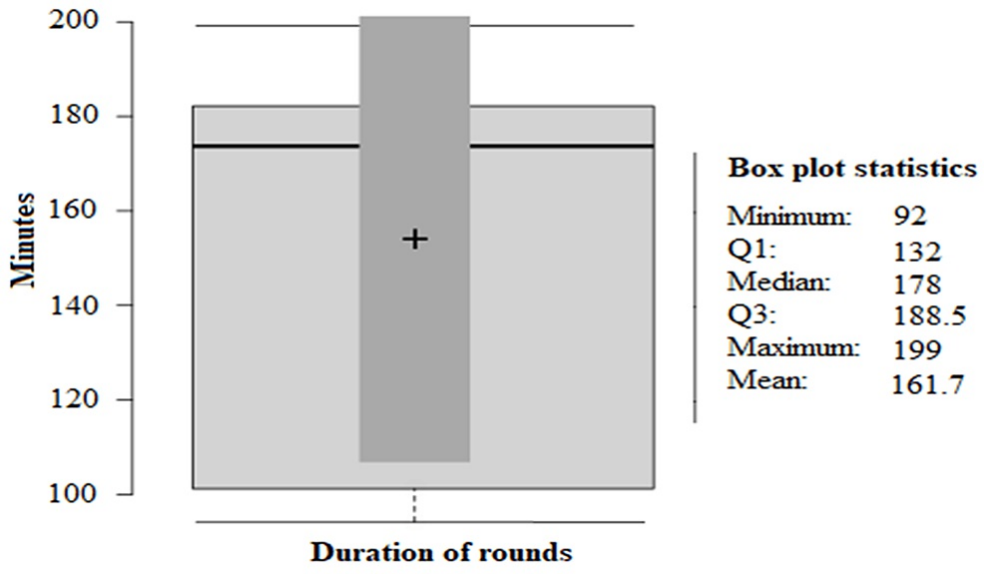
Duration of rounds in minutes.

The median total morning round time was 178 minutes, and the mean was 161.7 minutes (range = 92 to 199.5 minutes; SD = ±17.3). Figure 2 represents the round duration in minutes shown as a box plot.

**Figure 3:**
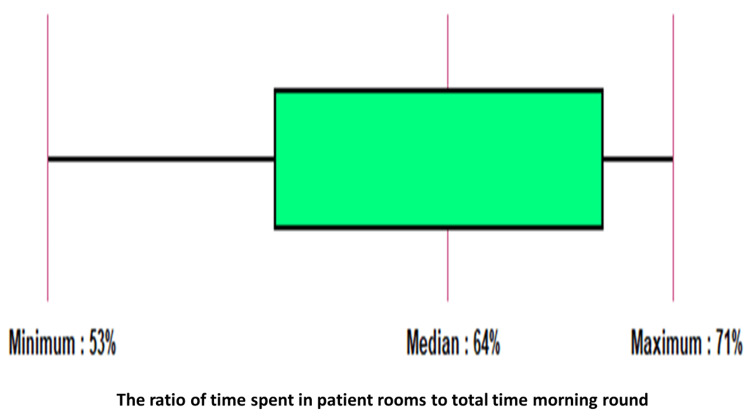
Ratio of time spent in patient rooms to the total morning rounds shown as a box plot.

A median of 64% (range = 53-71%) of the entire morning round time was spent in patient rooms. Figure 3 shows the ratio of time spent in patient rooms to the total morning rounds.

A total of 10 rounds of time-motion profiles were collected over the 10 rounds at three locations. The first location was the side of an inpatient room and the bed in the room. The second location was the hallway connecting the floors with stairs and corridors. The third location was the passage connecting the ED to the main entrance of HGH. Each day, the doctors went through the morning rounds for 35.7 minutes. The analysis found that 23.4% of the time was spent walking between patients in patient rooms, 40.1% in hospital corridors, and 35.6% in passageways to the emergency room. A description of the time physicians spent on morning rounds is given in Table 2.

**Table 2:** Description of the time physicians spent walking throughout morning rounds.

Walking inside the passageway	Mean motion time	Time distribution (%)
The mean amount of time spent walking between patients	8.7 ± 2.1 minutes	24.40%
The mean amount of time spent walking through hospital hallways	14.3 ± 4.5 minutes	40.10%
The mean amount of time spent walking through the hospital passageway to the emergency room	12.7± 3.4 minutes	35.60%
Average daily walking time spent by physicians on their morning rounds	35.7 ± 7.4 minutes	100.00%

Bedside visits lasted for an average of 161.7 ± 17.3 minutes. The doctor spent 7.1% of the time interrupted by requests for information and questions from clinic staff or relatives not present in the room, and 41.2% (66.7 ± 11.2 minutes) of time in direct patient contact, including physical examinations, examination results, nursing discussions, conversations with patients, and review of medications per round. The team members spent 11.4% (18.4 ± 4.1 minutes) of the round time interacting with electronic medical records and retrieving patients’ records from the system. Patients’ records included medical documents, laboratory information systems, radiological information systems, and microbiological information systems. Bedside teaching activities accounted for 18.26% (29.4 ± 5.4 minutes) of the total visiting time. More than one-fifth of the round time was spent walking between locations and patients (22.1%, 35.7 ± 7.4 minutes), and more than a quarter (29.4%, 47 ± 29.2 minutes) was spent on activities unrelated to patient care. Table 3 shows physicians’ time spent in various activities during the morning round.

**Table 3:** Physicians’ time spent on various activities during morning ward rounds.

Specific activities during morning rounds	Mean time spent	Time distribution (%)
Time spent on direct clinical matters	66.7 ± 11.2 minutes	41.20%
Time spent on teaching activities within the bedside	29.4 ± 5.4 minutes	18.20%
Time spent on the electronic health record	18.4 ± 4.1 minutes	11.40%
Time spent walking through the day’s morning rounds by physicians	35.7 ± 7.4 minutes	22.10%
Time spent on interruptions (interrupted by information requests and questions from clinical staff or family members who were not in the room)	11.5 ± 1.8 minutes	7.10%
The total time spent on morning rounds by consultants on the ward	161.7 ± 17.3 minutes	100.00%.

A team member walked an average of 763 ± 54.5 m (667- 872 m) per round, with an average round duration of 35.7 minutes (22.1%) of the total round time (161.7 ± 17.3 minutes). Even so, the walking distance varied with the patients’ location.

During the morning rounds, approximately 31.59% (241 mm) of the total walking distance was required to cross a passage to reach the ED from the hospital’s main entrance. This tour has 217 m between corresponding floors, representing 28.44% of the total distance of the round tour. The distance from the patient’s room to the hallway is 14.94% of the tour, and the distance from the hallway to the patient’s room is 191 m. Table 4 shows the average walking distance in meters and its percentage by location.

**Table 4:** Average walking distance in meters and its percentage according to location.

Location of the distance tracking system	Average walking distance	%
Distance between corresponding floors	217 ± 18.03 m	28.44%
Distance within room	114 ± 9.5 m	14.94%
Distance from outside of the corridor room in Hamad General Hospital	191 ± 15.9 m	25.03%
Distance between internal medicine and emergency medicine	241 ± 20.08 m	31.59%
Total	763 ± 54.50 m	100.00%

**Figure 4:**
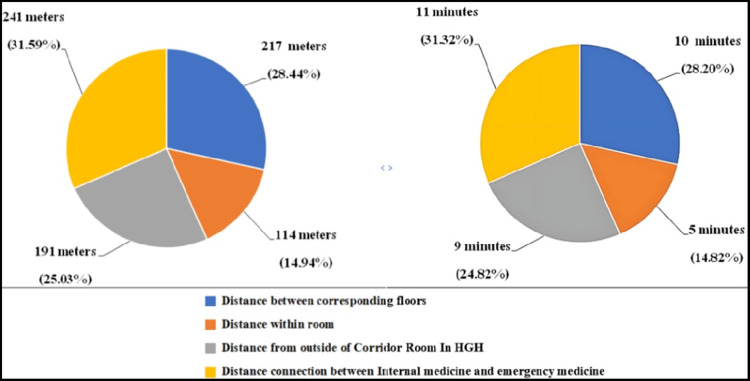
(A) Workplace displacement distribution and distance distribution between workplaces. (B) The pie graph represents the time spent on workplace displacement during the morning round. HGH: Hamad General Hospital

It was estimated that in 10 minutes, a team walked an average of 217 m between floors and 114 m inside the room for an average time of five minutes, for a total of 15 minutes. The average walking distance from the corridor outside the patients’ room was 191 m for an average time of nine minutes. In addition, the walking distance between the internal medicine main entrance and the emergency room main entrance was 241 m for an average time of 11 minutes. Figure 4 represents each time and percentage from the total round time.

## Discussion

This study outlined a model for the medical staff’s morning medical round activities in the general medical department at HGH. The usual daily activity during the round includes a brief medical history, new laboratory results, reviewing radiological results, discussing management plans, and education. Doing all these activities and rounding patients located in different places requires longer than rounding patients in nearby or one location. The time spent in achieving good patient care, educational activities, and walking distance between the patients and locations was assessed in this study.

It was noticed that the number of team members who attended the daily morning round varied with a mean of 8.6 members, similar to previously reported data (approximately nine healthcare workers) [12]. The morning round attendee numbers varied for several reasons, including night duties, sick leave, and other issues.

In the 10 rounds attended, the teams had 159 patients, with a median check-up time of 12 minutes per patient. This was consistent with previous findings from London NHS hospitals and Worthing Hospital in England [5,13]. In these two reports, patients were attended for 10-15 minutes during daily morning rounds, with an average regular visit time of 10 minutes and an average post-intake round time of 14 minutes per patient. This was consistent with what we observed after including the regular and post-intake rounds in our study. Studies have found that the median check-up time for each patient (patient encounter) varied significantly between different medical specialties. In geriatrics, oncology, surgery, intensive care medicine, neurology, pediatrics, and rehabilitation wards, direct patient contact lasted 16 minutes, 15 minutes, 12 minutes, 14 minutes, 9 minutes, 10 minutes, and 19 minutes, respectively [1,14-19]. Emergency admission of complicated cases, unplanned admissions, and re-hospitalizations of discharged patients could cause this difference in patient check-up times in general medical wards [20].

Furthermore, the internists in our medical department manage patients with a multidisciplinary approach, including other specialties such as endocrinology, rheumatology, nephrology, and gastroenterology. Furthermore, patients in the medical department usually have more than clinical diseases (diabetes, hypertension, chronic obstructive pulmonary disease, pneumonia, heart failure, and stroke), and the patients are usually elderly (over 60% older than 50 years) [21]. In this study, the average round duration was 161.7 minutes, significantly longer than the 105 and 122 minutes recorded in previous studies [1]. This was due to the time spent moving between patients in the emergency department and HGH wards, accounting for 35.60% (35.7 minutes) of the total movement time.

There is no doubt that patient room visits play a crucial role in determining the total round time, accounting for 70.80% of the total daily morning round time. The medical team must meet a specific deadline because the conversation with the patient can differ depending on the patient, family members, and staff members. Previous research showed that physicians spent more time introducing patients to students [15]. Pre-rounding with information about the patients and their diseases and determining the external factors influencing the doctor-patient attendance time are the best strategies to reduce round time without compromising patient safety and care [16]. However, more supportive research is needed to determine if these factors can shorten the length of the doctor-patient conversation without compromising patient care and safety. Other factors such as social and economic status, ethnicity, gender, educational level, and disruption can significantly affect the doctor-patient contact time. All these factors were thought to have played a role, leading to longer rounding times in the general medicine department.

Interestingly, despite the increase in the number of admissions reported, the doctor-patient contact was not affected, as reported in this study. However, the number of admissions and the dispersion of hospitalization locations increased the morning round time. Observers noted that some residents followed patients, revised test results, prepared case summaries, and ordered new tests before morning rounds to save the round time. When this approach was implemented, students appreciated and felt optimistic and reported that they benefited more because they had more time to discuss their cases; however, this approach still cost an additional 29.4 minutes (18.20%) of the total round time. Prioritizing tasks, such as starting with a new patient and consulting critically ill patients before stable patients, were maneuvers used to reduce round time; however, they did not positively impact productivity.

During the attended daily morning round, activities including discussing patient investigation results, diagnosis, and care plans and explaining to patients about medications, plans, results of the investigations, disposition to other facilities, and medical education contributed to the lengthening of the ward round [17]. We observed that the number of patients differed between the teams depending on the number of new admissions and the number of patients the team had. Furthermore, it was noticed that some family members came in between and asked about their patient’s condition and therapy plan, which affected the daily morning round rhythm and led to more time wastage.

During the round, clinical matters focused on understanding the key issues, diagnosing them, and treating them promptly to achieve the best possible outcome. Based on the study’s results, an average of 66.7 minutes (41.2%) of the total round time was spent on direct clinical matters, consistent with previous studies [19,20]. Interestingly, it was noticed that the round time duration varied between teams mostly due to non-medical issues rather than medical care issues delivered to patients and their family members.

In addition, 18.20% (approximately 29.4 minutes) of the bedside time was spent on educational activities with medical students and residents, which was longer than that reported by a previous study [20]. Teaching during clinical rounds is important for teaching medical students despite the new advanced clinical skills laboratories and simulation-based medical education. Time spent teaching students and interns during the daily morning rounds was needed to ensure that they received prompt feedback and tracked their progress throughout the educational program. Accordingly, students, interns, and attendees should be involved actively in the round process to enhance learning as much as possible and to gain bedside experience [21]. Based on informal conversations and small talks with most team clinicians, patient interaction benefited students and residents and improved their clinical practice.

Electronic medical records (EMR) enable physicians to spend more time with patients and improve the quality of care. EMR of patient data, including patient family, personal, medical, allergy, surgical, social, laboratory, diagnostic, and treatment information, which was digitally captured, took only 18.4 minutes (10%) of the round time, which was less than previously reported studies [22,23]. This improvement in retrieving patient data electronically improved the daily morning time [24,25].

Interruption with teams during the round led the team member(s) to spend more time dealing with issues unrelated to patient medical care. Interruption during patient care negatively impacts the round process, compromising patient safety, and may lead to clinical errors and low-profile patient safety [26]. A study investigating round activities and simultaneous workflow interruptions found that doing different tasks, such as administrative tasks, during the round affected the round time [25]. Our study found that 8.60% of round duration was due to interruptions by face-to-face conversations, telephone calls, instant messaging, and overhead communications. Disruption duration can be reduced by limiting visitors and having a unidirectional entrance and exit point [27,28].

During the study, participants walked an average of 35.7 minutes, with 21.10% walking time. Comparative data are very few; however, a previous study reported a median of 24.9 minutes [29]. Due to the long distance walked and the increased disruption rates, the time wasted was more than previously reported. Hence, locating patients’ rooms in one area or nearby areas and implanting one entry and exit point (unidirectional) will decrease interruption and reduce wasted time.

This study found that the most accurate movement time was provided by round teams walking in the stationary room at the end of the round [30]. The study also found that the time spent moving around the patient’s rooms in one location and between patients was minimal or even optimal, accounting for 14.8% of the total movement time between four and 13 minutes. A large proportion of the time was spent in the passageways connecting the emergency department and HGH patient locations, accounting for 31.32% (11 minutes). Compared to three previous studies [31-33], the movement time averaged 6.6% and ranged from 4.2% to 77%. Our study reported a shorter time (21.10%) spent. Additionally, it has been noticed that most of the round’s time spent outside the patient’s room accounted for 85.18% of the walking time (equivalent to 30.7 minutes). According to these results, to reduce wasted time due to long distances, internal medicine patient beds should be relocated to one area or other nearby areas with a one-way passage. Furthermore, it was noticed that some rooms had one or two beds instead of four beds. Filling beds in one room reduces walking distance and time wastage.

This study also found that round team members traversed an average of 763 m (667-872 m) during their rounds, which could cause physical stress and reduce effectiveness and concentration, affecting team performance, patient care management, safety, and fairness [34]. Therefore, reducing the distance doctors travel daily allows team members to spend more time with their patients and improve the quality and cost of care [35]. Health, quality, and safety leaders recommend that healthcare providers better use the short passageway during daily rounds. This saves time, reduces fatigue, increases productivity, and allows staff to spend more time with patients. A published report examined hospitals’ and colonics’ designs affected the nurses’ and doctors’ walking distance and its effect on performance and time waste. The study advised that hospital building layouts and designs should consider the distances between areas to reduce walking distance and time wastage to improve the performance efficiency of the staff. Many variables, including the number of private rooms, the distribution of nursing rooms, and the ward layout, determine the distance between rooms in the wards.

Additionally, the number of physicians and nurses attending, patient type and the complexity of their disease(s), and the round time of the day should be considered [36]. All of these factors generally affect the round time and must be considered to achieve the recommended round time. A study on the highest acceptable distance for nurses reported that nurses are expected to walk about 3.3 miles during an eight-hour day shift [37]. In this study, we did not examine this issue, despite its importance, because the study aimed to evaluate round times and possible solutions to reduce them. Furthermore, nurses attended the round in their unit, and there were no nurses with the team while they moved between the different locations. However, it is important to study caregivers’ walking distances and the time they spend walking the distances, as addressing these issues will improve performance and the quality of care.

In our informal conversations with the team members about improving the daily morning time in the medical department at HGH, most team members agreed that the round time was long and agreed to have up-to-date plans that aim to decrease the morning daily round waste time. Some team members believed that the medical round is not much valued, but it is challenging to demonstrate its value rather than discuss its importance. Making wise decisions to get the best results is crucial.

The main limitation was the quality of rounds measured by direct observation without considering the perspective of health management or health economics (a new scientific field called visit economics). In addition, the characteristics of physicians and practices were not compared in this study because the data were collected at one facility and may not apply to other contexts. In addition, data were collected for post-admission and follow-up rounds. The data size was small (heterogeneous data), limiting the findings of duration to medians rather than the average time, which may reduce data validity. Another area for improvement of our study was the time frame used for data collection, which was during the summer season (specifically from July 1 to July 30, 2022). This period corresponded to the residents’ rotation after completing a year of training, which provided them with more experience with admission requirements and reduced the number of unnecessary admissions. In comparison, in wintertime, many citizens travel for vacation during study time, which could affect the number of admissions and the round time.

## Conclusions

The general internal medicine department morning round takes more time than expected. The longer morning rounds are due to teaching, patient/family conversations, disjointed discussions, and exceptionally long round-tour walking. This can compromise patient safety and efficacy. Grouping patients in one area and creating a one-way geographic location are recommended to save time and minimize disruption. A repeat study in the same department would be required to assess the impact of these recommendations on the general internal medicine round times.
